# Research trends and areas of focus on cryoablation and oncology: A bibliometric analysis from 2001 to 2020

**DOI:** 10.1097/MD.0000000000032513

**Published:** 2022-12-30

**Authors:** Hang Liu, Changen Song, Bingzhe Zhang, Rong Luo, Jijin Yang

**Affiliations:** a Department of interventional therapy, Changhai Hospital, Navy Medical University, Shanghai, China; b Department of radiology, Shanghai Shidong Hospital of Yangpu District, Shanghai, China.

**Keywords:** bibliometric analysis, citations, cryoablation, tumor

## Abstract

Cryoablation is an interdisciplinary, widely used treatment approach for several types of solid tumors, making it difficult to obtain a comprehensive picture of its current status and popular research topics. This study aimed to use a bibliometric approach to understand important research themes and trends in cryoablation and oncology.

**Methods:**

Literature studies on cryoablation and oncology from 2001 to 2020 were extracted from the Web of Science. A bibliometric analysis was performed based on the annual publication volume, several journal articles and local citation score, and distribution of keywords and trends in the literature using tools such as COOC version 9.94, VOSviewer version 1.6.17, and the bibliometrix version 3.1.3 R package.

**Results:**

This study included 2793 publications. Total yearly publications have plateaued over the last 20 years. Five research themes were presented in the keyword network, including clinical applications of cryoablation in liver, lung, kidney, prostate, and skin cancers and comparison of cryoablation with other energy ablations. After 2012, 2 new research topics emerged: synergy between cryoablation and immunotherapy in tumors and cryoablation of Barrett esophagus. The high cited literatures are dominated by studies related to cryoablation for renal and prostate cancer treatment, but they also reflect the recent increasing interest in immunotherapy and bone metastases. Twenty important journals were identified, with *Cryobiology* publishing the most articles.

**Conclusion:**

Bibliometric analysis of studies related to tumor cryoablation can help researchers rapidly comprehend popular topics and determine future trends, guiding future research directions.

## 1. Introduction

Thermal ablation is a treatment method for patients with various localized tumors. Cryoablation has several advantages over other thermal ablation procedures, including the anesthetic effect of hypothermia, effective visibility of the ablation area in imaging devices, preservation of fibroblasts, and activation of the immune system.^[1,2]^ Moreover, this procedure can treat tumors in almost all organs. Thus, there are a large number of studies on cryoablation in the field of oncology. However, previous literature has only focused on the application of cryoablation in specific tumor types.^[3,4]^ It has not addressed important issues of hotspots and cryoablation trends in the field of oncology on long-term scales. This causes major difficulties in achieving a comprehensive understanding of the fields of cryoablation and oncology.

Bibliometric analysis can qualitatively and quantitatively explore knowledge structures and generate trends in specific research fields.^[5]^ It can also help researchers to effectively integrate information in major scientific publications and identify classic literature or research hotspots.^[6,7]^ More recently, there have been an increasing number of publications focusing on applying bibliometric analysis in the medical field.^[8–10]^ However, using bibliometric analysis, only a few previous studies have analyzed growth trends of publications, publishing sources, keywords, and topics in cryoablation and oncology.^[11,12]^

This study aimed to characterize the current research status of cryoablation and oncology and predict hot topics in the future using bibliometrics.

## 2. Materials and methods

### 2.1. Data sources and search strategies

Bibliometric indicators of published items were retrieved from the Science Citation Index-Expanded and Conference Proceedings Citation Index-Science databases in the Web of Science (WoS) Core Collection of Clarivate Analytics on November 17, 2021. We conducted several topic searches (TS) using the following search strategies: #1 TS = (cancer) OR TS = (neoplasm) OR TS = (tumor) OR TS = (neoplasia) OR TS = (carcinoma) OR TS = (sarcoma) OR TS = (malignancy); #2 TS = (cryoablation) OR TS = (cryosurgery) OR TS = (cryotherapy); #3 TS = (“cold destruction”) OR TS = (“cold ablation”) OR TS = (“cold therapy”) OR TS = (“freeze therapy”) OR TS = (“freeze ablation”) OR TS = (“freezing ablation”) OR TS = (“freezing therapy”) OR TS = (“freeze treatment”) OR TS = (“freezing treatment”) OR TS = (“cold treatment”) OR TS = (cryotreatment); #4 (#2) OR (#3); #5 (#4) AND (#1). The data used in this study were obtained from the WoS database; hence, ethical approval does not apply to this study.

### 2.2. Data screening and preprocessing

**Figure 1.**
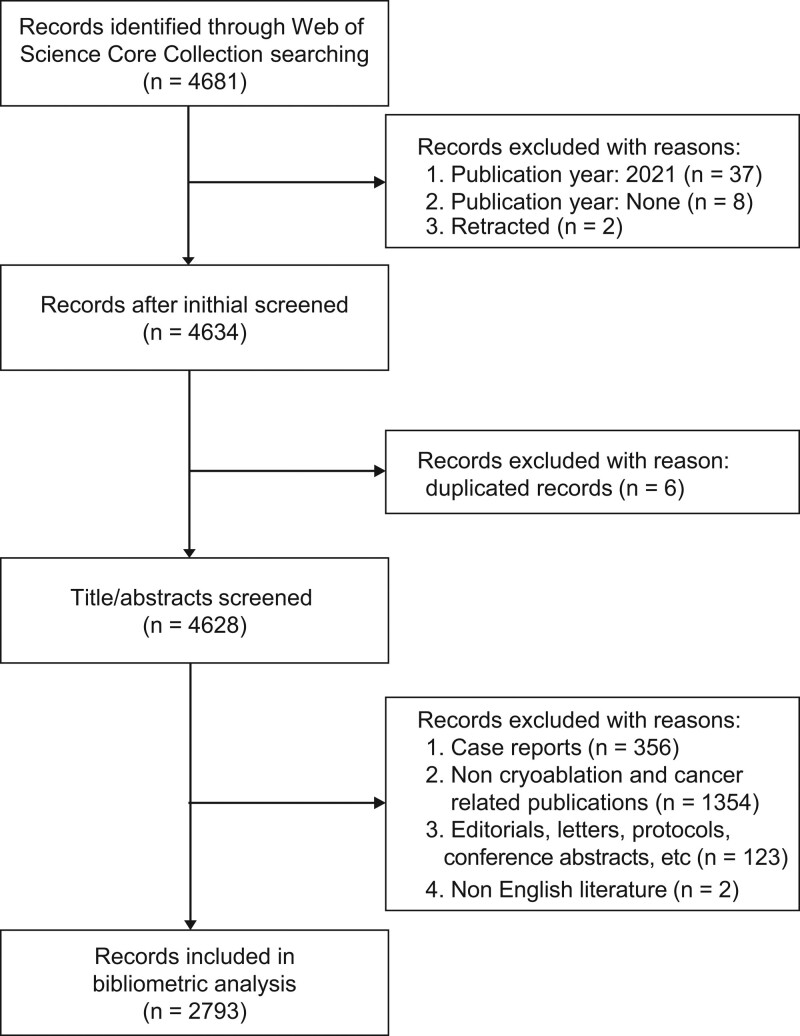
Flow diagram of the literature selection process.

Document types were limited to “article,” “review,” and “proceedings paper.***”*** The publication period was restricted to the period from 2001 to 2020. Only publications in English were included. The exclusion criteria were publications in the veterinary field; irrelevant publications; case reports or case series with less than 5 cases; “editorial notes,” “conference proceedings,” “letters,” “protocols,” “books,” and “book series”; duplicated or retracted publications; publications published after 2020; and publications in nonEnglish languages. Two investigators reviewed the bibliometric indicators of the publications independently. If there were disagreements, they were discussed and resolved with the help of a third investigator with more than 10 years of experience in cryoablation and oncology. The screening process is shown in Fig. 1.

To further evaluate the screened data set, the contents of the “author keywords (DE)” and “keywords plus (ID)” columns were combined into a new column named “DEID” column. *Keywords plus* were retrieved from the titles of publications cited by authors in the Clarivate Analytics database.^[13]^ Synonym conversion of the keywords from the “DEID” column was performed using text from the processing software COOC version 9.94.^[14]^ Subsequently, all duplicated and meaningless keywords in each publication were deleted.

### 2.3. Bibliometric analysis

Analyses were performed using VOSviewer (version 1.6.17, Leiden University, Netherlands) and R package bibliometrix (version: 3.1.3) in R (version 4.1.0).^[15]^ Annual and cumulative numbers were calculated to construct growth models of the publication output.

Local citation score (LCS) was used to evaluate the impact and creativity of a publication. LCS refers to the number of times other publications cited one publication in a specific data set. Although both LCS and global citation score (GCS) can represent the impact and creativity of one research, there is a contradiction between the 2 indicators in interdisciplinary fields. This is because citations from researchers in other fields was beneficial to GCS of literature, but had less effect on LCS. It also illustrates that the impact ability of these publications is limited in the specific field. Therefore, compared to GCS, LCS is less susceptible to other factors and more representative than GCS when exploring influential literature in the field of cryoablation and neoplasm—which is a subdivision of interventional oncology—is required. Considering the effects of publication year, we further calculated the average annual LCS (total LCS/publication year), actual annual LCS, and total actual annual LCS in the recent 3 years (2018–2020).

The productive publication sources of cryoablation and oncology were identified using Bradford law.^[16,17]^ Bradford law can be used to identify the “core” journals in one field. According to this law, all publication sources were arranged in descending order by their respective number of published articles. Moreover, they were divided into 3 zones: core, middle, and minor zones, where each zone published one-third of the total publications. These zones signify the weight of publication sources in the field. The number of publication sources in the core zone was the lowest, whereas citations of articles in these sources were relatively high. The annual publication number and LCS were also used to evaluate those publication sources.

Moreover, high-frequency keywords (>50) were extracted to construct a keyword cooccurrence network. The co-occurrence data were normalized by equivalence index to calculate a similarity measure and clustered using leading eigenvector algorithms. Subsequently, the network was graphically visualized using the VOSviewer software. Finally, we used thematic maps to quantify and visualize the thematic evolution of the cryoablation and oncology fields over 20 years from 2001 to 2020.^[18,19]^ All keywords of each period were included, except rare keywords. The 20 years were divided into 2 periods, with 2012 being the dividing point. The X-axis of the thematic maps represents the degree of relevance (centrality), which measures the strength of interaction of a cluster network and others in the same map. The Y-axis of the thematic maps represents the degree of development (density), which measures the strength of interaction of all keywords within a cluster network.

## 3. Results

### 3.1. Analysis of scientific publication output

**Figure 2.**
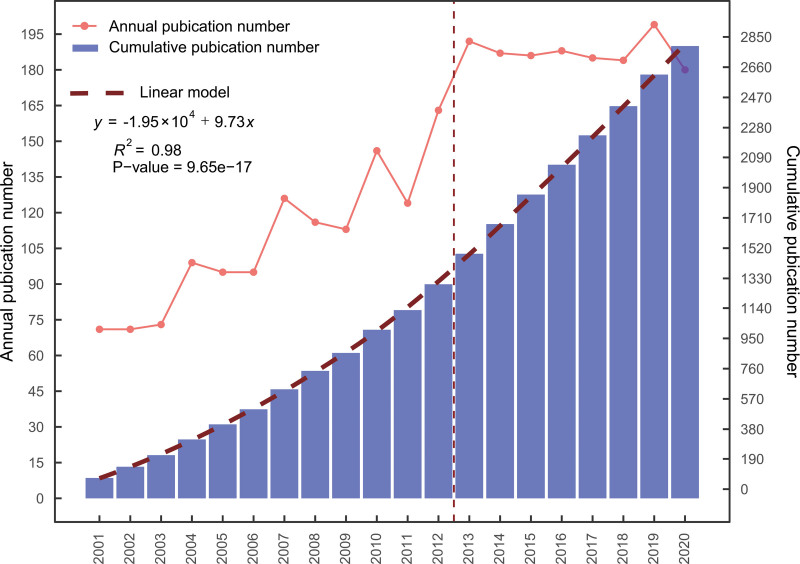
Annual and cumulative publication outputs on cryoablation and oncology research from 2000 to 2020.

We initially identified 4681 scientific productions using a predesigned retrieval strategy. Based on the exclusion criteria, 2793 scientific productions were included in the bibliometric analysis (Fig. 1). The number of annual and cumulative publications in the research area of cryoablation and oncology from 2001 to 2020 is displayed in Fig. 2. For almost 20 years, the literature on cryoablation and oncology has been steadily increasing. From 2001 to 2012, the annual number of publications increased from 71 to 163, showing a wavelike pattern, whereas, over the subsequent 8 years, the annual number of publications remained unchanged, showing a horizontal linear pattern at approximately 190 annually. Moreover, the growth model of the cumulative number of publications from 2001 to 2020 fits a linear growth model (*R*^2^ = 0.98, *P*-value < .001).

### 3.2. Analysis of keywords and topics

Keywords, including author keywords and keywords plus (provided by WoS), of one publication tended to map to its topic.^[20]^ Researchers can evaluate the foci and hotspots of research fields by learning the distribution of keywords. After data processing, 5986 keywords (total word frequency, 29,615) were included in the present analysis. Rare keywords (total frequency, 1) accounted for 66.52% of the total keywords, whereas the word frequency only accounted for 15.61% of the total word frequency. The word frequency of the top 900 keywords (15.6% of all keywords) accounted for 74.9% of the total word frequency (see Fig. S1, Supplemental Digital Content, http://links.lww.com/MD/I227, which illustrates the distribution pattern of all keywords). Hence, only a few keywords are required to evaluate the research field.

**Figure 3.**
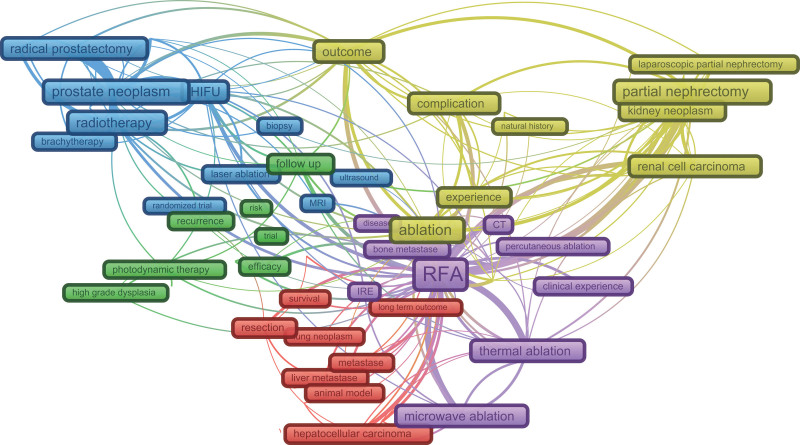
The co-occurrence network of high-frequency keywords. In total, 5 clusters were identified: “clinical value of treating liver/lung neoplasm with cryoablation (in red),” “clinical value of treating skin/mucosa neoplasm with cryoablation (in green),” “clinical value of treating prostate neoplasm with cryoablation (in blue),” “clinical value of treating renal neoplasm with cryoablation (in yellow),” and “comparison of different ablation procedures on neoplasm (in purple).” Note: High-frequency refers to the frequency of keywords >50.

High-frequency keywords (n = 90) with a frequency >50 were selected to construct a keyword co-occurrence network (Fig. 3). The networks illustrated that 90 keywords were grouped into 5 clusters, which were defined as “clinical value of treating liver/lung neoplasm with cryoablation,” “clinical value of treating skin/mucosa neoplasm with cryoablation,” “clinical value of treating prostate neoplasm with cryoablation,” “clinical value of treating renal neoplasm with cryoablation,” and “comparison of different ablation procedures on neoplasm.” The cluster of “clinical value of treating liver/lung neoplasm with cryoablation” (cluster one, red) contained 24 keywords. “Liver neoplasm,” “metastases,” “lung neoplasm,” “resection,” “survival,” and “arterial chemoembolization” were the typical representative keywords of cluster one. The cluster of “clinical value of treating skin/mucosa neoplasm with cryoablation” (cluster 2, green) contained 20 keywords, the typical representative keywords of which were “photodynamic therapy,” “high-grade dysplasia,” “basal cell carcinoma,” “cervical neoplasm,” “recurrence,” and “follow-up.” Within the cluster of “clinical value of treating prostate neoplasm with cryoablation” (cluster 3, blue), there were 18 keywords. The typical representative keywords were “prostate neoplasm,” “radiotherapy,” “brachytherapy,” “radical prostatectomy,” “high-intensity focused ultrasound (HIFU),” “local therapy,” and “quality of life.” In the cluster “clinical value of treating renal neoplasm with cryoablation” (cluster 4, yellow), there were 16 keywords. The typical representative keywords included “kidney neoplasm,” “laparoscopic,” “nephrectomy/partial nephrectomy,” “outcome,” and “complication.” In the cluster of “comparison of different ablation procedures on neoplasm” (cluster 5, purple), there were 12 keywords. The typical representative keywords were “radiofrequency ablation (RFA),” “microwave ablation (MWA),” “irreversible electroporation (IRE),” “thermal ablation,” “image-guided,” “clinical experience,” and “long-term outcome.”

**Figure 4.**
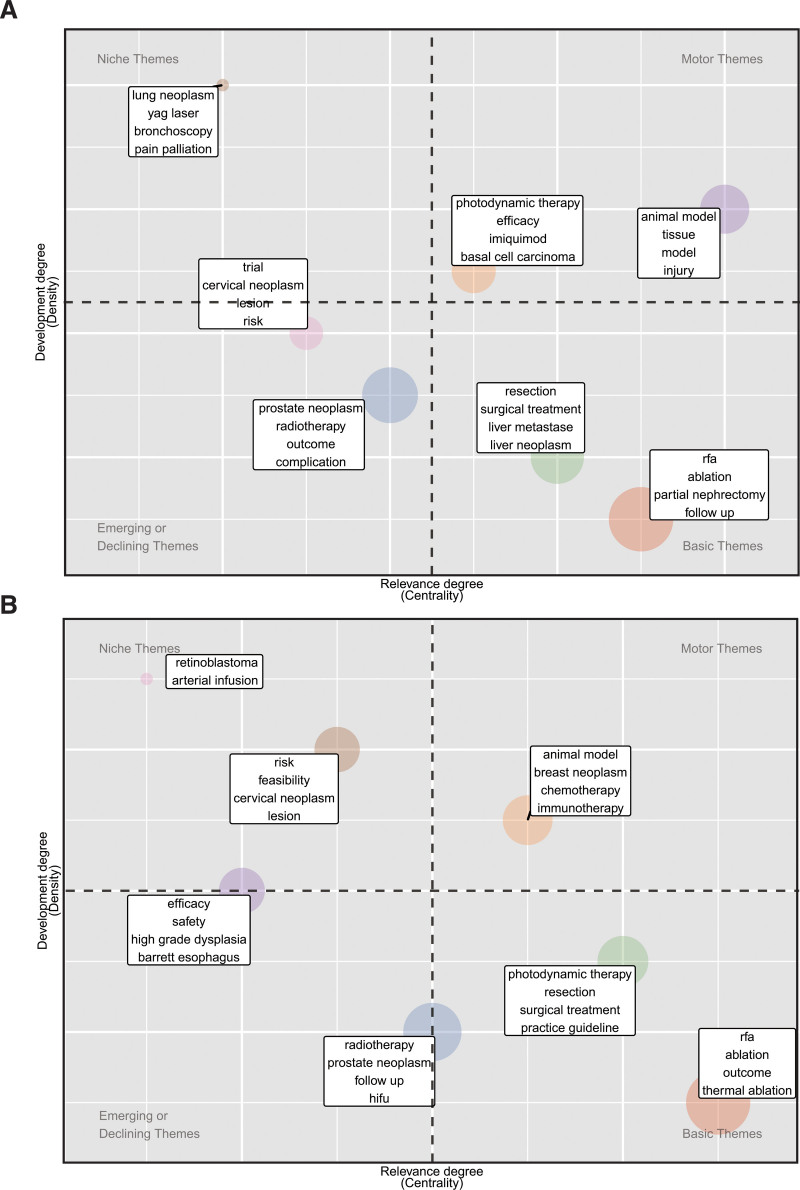
Temporal evolution of topics on cryoablation and oncology research. (a) Thematic map of keywords’ network clusters from 2001 to 2012. (b) Thematic map of keywords’ network clusters from 2013 to 2020. Each bubble represents a cluster of keywords, and a bubble label is a group of words with the highest occurrence rate.

Based on the co-occurrence networks of high-frequency keywords, thematic maps were further built to explore the importance and development of themes. Figures 4a and 4b show that different clusters were noted within 2 periods: 2001–2012 and 2013 to 2020. Seven clusters were identified according to relevance and degree of development by analysis of keywords of publications from 2001 to 2012 (Fig. 4a). The upper right quadrant, also named motor themes, representing high density and centrality, contained 2 clusters. One cluster included “animal model,” “tissue,” “model,” and “injury,” whereas another cluster included “photodynamic therapy,” “efficacy,” “imiquimod,” and “basal cell carcinoma.” In the lower right quadrant, also named basic themes, 2 clusters were characterized by high centrality but low density. The labels of one cluster were “resection,” “surgical treatment,” “liver metastases,” and “liver neoplasm,” whereas the labels of the other one included “RFA,” “ablation,” “partial nephrectomy,” and “follow-up.” Only one cluster was in the upper left quadrant (niche themes), and its labels were “lung neoplasm,” “YAG laser,” “bronchoscopy,” and “pain palliation.” Finally, 2 clusters were positioned in the lower left quadrant (emerging or declining themes) with low centrality and density. One cluster included “trial,” “cervical neoplasm,” “lesion,” and “risk,” whereas the other one included “prostate neoplasm,” “radiotherapy,” “outcome,” and “complication.”

Topics in the field of cryoablation and tumor research in the thematic map have evolved during the study period. Although there were also 7 clusters in the thematic map from 2013 to 2020, the characteristics of each cluster changed (Fig. 4b). There was only one cluster in motor themes, and its representative keywords included “animal model,” “breast neoplasm,” “chemotherapy,” and “immunotherapy.” Two new clusters, characterized by low centrality, were located at the niche themes and the border between niche themes and emerging or declining themes. Labels of the cluster in the niche themes were “retinoblastoma” and “arterial infusion,” and those of another emerging cluster included “efficacy,” “safety,” “high-grade dysplasia,” and “Barrett esophagus.” Moreover, the density of the cluster labeled “cervical neoplasm” and “risk” increased, and one label among the labels of the cluster representing “prostate neoplasm” changed into “HIFU.” In addition, the characteristics of 2 other clusters positioned in basic themes also changed slightly. The cluster used to contain “resection” and “surgical treatment” included 2 new keywords: “photodynamic therapy” and “practice guideline” in place of “liver neoplasm” and “liver metastases.” The labels of the cluster that contained “RFA,” “ablation,” and “follow-up” changed into a similar collection containing “RFA,” “ablation,” “outcome,” and “thermal ablation.”

### 3.3. Analysis of highly cited publications

**Figure 5.**
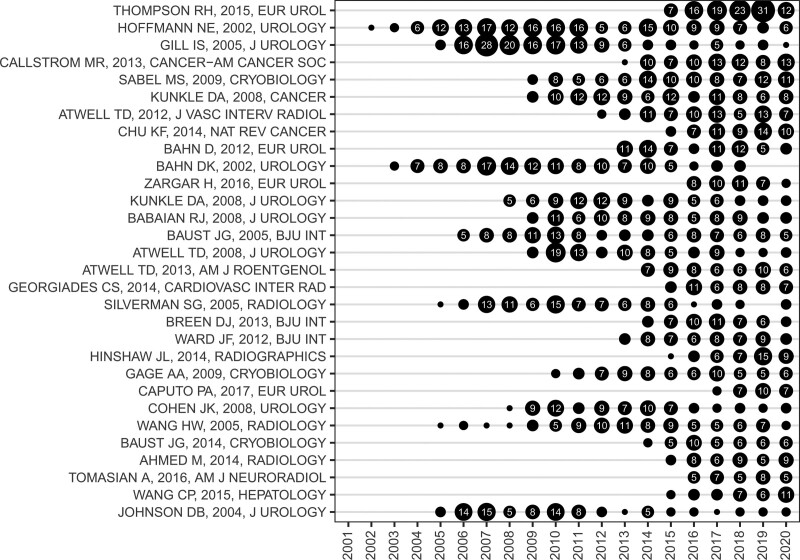
Actual annual LCS of the top 30 literature ranked by average annual LCS. The longitudinal axis indicates the author’s name, publication year, and journal of the literature. Note: the size of the black dots is proportional to the actual annual LCS of the literature. Numbers inside black dots indicate the actual annual LCS of the literature. LCS = local citation score.

LCS has been used to objectively demonstrate the degree of attention on publications in a specific field. As the analysis of citations also needs to consider the skewed distribution of citations,^[21]^ we ranked all publications included in this analysis according to the average annual LCS to preclude the interference of the time of publication on the results. A detailed list of the top 30 publications by average annual LCS is provided in Table 1. The topics of these publications included the following: application of cryoablation for renal or prostatic neoplasm, injury mechanisms of cryoablation, and effects of cryoablation on the immune system. Moreover, a few publications focused on applying cryoablation for liver neoplasm or bone metastasis. Figure 5 shows the actual annual LCS of the top 30 publications by average annual LCS. The actual annual LCS often reflects the life cycles of literature and demonstrates which publications are affecting a research field over time.^[22]^ The actual annual LCS per publication peaked within 3 to 5 years after publication, followed by a decline after maintenance for some years. In all literature published for over 10 years, obsolescence was observed in all the included studies, except for one, Cryoimmunology: a review of the literature and proposed mechanisms for stimulatory versus suppressive immune responses. The total LCS in 2018–2020 (3-year LCS) was calculated to reveal the prime attention of investigators in recent years (Table 2). The top 5 publications were a Comparison of partial nephrectomy and percutaneous ablation for cT1 renal masses (3-year LCS, 66), Percutaneous image-guided cryoablation of painful metastases involving bone: multicenter trial (3-year LCS, 33), Thermal ablation of tumors: biological mechanisms and advances in therapy (3-year LCS, 33), Percutaneous tumor ablation tools: microwave, radiofrequency, or cryoablation-what should you use and why? (3-year LCS, 31), and Cryo-immunology: a review of the literature and proposed mechanisms for stimulatory versus suppressive immune responses (3-year LCS, 30).

**Table 1:** Top 30 publications of average annual LCS.

DOI	PY	LCS_PY	LCS	GCS	TI
10.1016/j.eururo.2014.07.021	2015	15.43	108	257	Comparison of partial nephrectomy and percutaneous ablation for cT1 renal masses
10.1016/S0090-4295(02)01683-7	2002	9.1	182	357	The cryobiology of cryosurgical injury.
10.1097/01.ju.0000158154.28845.c9	2005	8.65	147	296	Renal cryoablation: outcome at 3 years
10.1002/cncr.27793	2013	8.22	74	165	Percutaneous image-guided cryoablation of painful metastases involving bone: multicenter trial
10.1016/j.cryobiol.2008.10.126	2009	7.69	100	174	Cryo-immunology: a review of the literature and proposed mechanisms for stimulatory versus suppressive immune responses
10.1002/cncr.23896	2008	7.29	102	274	Cryoablation or radiofrequency ablation of the small renal mass: a meta-analysis
10.1016/j.jvir.2011.09.008	2012	7.2	72	140	Complications following 573 percutaneous renal radiofrequency and cryoablation procedures
10.1038/nrc3672	2014	6.75	54	893	Thermal ablation of tumors: biological mechanisms and advances in therapy
10.1016/j.eururo.2012.03.006	2012	6.7	67	156	Focal cryotherapy for clinically unilateral, low-intermediate risk prostate cancer in 73 men with a median follow-up of 3.7 years
10.1016/S0090-4295(02)01678-3	2002	6.45	129	199	Targeted cryoablation of the prostate: 7-year outcomes in the primary treatment of prostate cancer
10.1016/j.eururo.2015.03.027	2016	6.33	38	75	Cryoablation for Small Renal Masses: Selection Criteria, Complications, and Functional and Oncologic Results
10.1016/j.juro.2007.11.047	2008	6.14	86	367	Excise, ablate or observe: the small renal mass dilemma--a meta-analysis and review
10.1016/j.juro.2008.07.108	2008	6.14	86	149	Best practice statement on cryosurgery for the treatment of localized prostate cancer
10.1111/j.1464-410X.2005.05502.x	2005	6.12	104	191	The molecular basis of cryosurgery
10.1016/j.juro.2008.01.144	2008	5.93	83	127	Percutaneous renal cryoablation: experience treating 115 tumors
10.2214/AJR.12.8618	2013	5.78	52	127	Percutaneous ablation of renal masses measuring 3.0 cm and smaller: comparative local control and complications after radiofrequency ablation and cryoablation
10.1007/s00270-013-0831-8	2014	5.5	44	84	Efficacy and safety of percutaneous cryoablation for stage 1A/B renal cell carcinoma: results of a prospective, single-arm, 5-year study
10.1148/radiol.2362041107	2005	5.41	92	154	Renal tumors: MR imaging-guided percutaneous cryotherapy--initial experience in 23 patients
10.1111/bju.12122	2013	5.33	48	71	Percutaneous cryoablation of renal tumors: outcomes from 171 tumors in 147 patients
10.1111/j.1464-410X.2011.10578.x	2012	5.3	53	116	Focal cryotherapy for localized prostate cancer: a report from the national Cryo On-Line Database (COLD) Registry.
10.1148/rg.345140054	2014	5.25	42	164	Percutaneous tumor ablation tools: microwave, radiofrequency, or cryoablation--what should you use and why?
10.1016/j.cryobiol.2009.10.001	2009	5.23	68	153	Experimental cryosurgery investigations in vivo
10.1016/j.eururo.2016.08.039	2017	5.2	26	52	Cryoablation versus Partial Nephrectomy for Clinical T1b Renal Tumors: A Matched Group Comparative Analysis
10.1016/j.urology.2007.09.059	2008	5.14	72	103	Ten-year biochemical disease control for patients with prostate cancer treated with cryosurgery as primary therapy
10.1148/radiol.2351030747	2005	5.06	86	149	Thoracic masses treated with percutaneous cryotherapy: initial experience with more than 200 procedures
10.1016/j.cryobiol.2013.11.001	2014	5	40	75	Mechanisms of cryoablation: clinical consequences on malignant tumors
10.1148/radiol.14132958	2014	5	40	557	Image-guided tumor ablation: standardization of terminology and reporting criteria--a 10-year update
10.3174/ajnr.A4521	2016	5	30	72	Spine cryoablation: pain palliation and local tumor control for vertebral metastases
10.1002/hep.27548	2015	4.86	34	125	Multicenter randomized controlled trial of percutaneous cryoablation versus radiofrequency ablation in hepatocellular carcinoma
10.1097/01.ju.0000135833.67906.ec	2004	4.83	87	194	Defining the complications of cryoablation and radio frequency ablation of small renal tumors: a multi-institutional review

DOI = digital object unique identifier, GCS = global citation score, LCS = local citation score, LCS_PY = local citation score/(2022 - PY), PY = publication year, TI = title of literatures.

**Table 2:** Top 30 publications of total LCS in 2018-2020.

rank	DOI	LCS_3 years		TI
1	10.1016/J.EURURO.2014.07.021		66	Comparison of partial nephrectomy and percutaneous ablation for cT1 renal masses
2	10.1002/CNCR.27793		33	Percutaneous image-guided cryoablation of painful metastases involving bone: multicenter trial
3	10.1038/NRC3672		33	Thermal ablation of tumors: biological mechanisms and advances in therapy
4	10.1148/RG.345140054		31	Percutaneous tumor ablation tools: microwave, radiofrequency, or cryoablation--what should you use and why?
5	10.1016/J.CRYOBIOL.2008.10.126		30	Cryo-immunology: a review of the literature and proposed mechanisms for stimulatory versus suppressive immune responses
6	10.1016/J.JVIR.2011.09.008		25	Complications following 573 percutaneous renal radiofrequency and cryoablation procedures
7	10.1002/HEP.27548		24	Multicenter randomized controlled trial of percutaneous cryoablation versus radiofrequency ablation in hepatocellular carcinoma
8	10.1016/J.EURURO.2016.08.039		24	Cryoablation versus Partial Nephrectomy for Clinical T1b Renal Tumors: A Matched Group Comparative Analysis
9	10.1007/S00270-013-0831-8		23	Efficacy and safety of percutaneous cryoablation for stage 1A/B renal cell carcinoma: results of a prospective, single-arm, 5-year study
10	10.1148/RADIOL.14132958		23	Image-guided tumor ablation: standardization of terminology and reporting criteria--a 10-year update
11	10.1002/CNCR.23896		22	Cryoablation or radiofrequency ablation of the small renal mass: a meta-analysis
12	10.2214/AJR.12.8618		22	Percutaneous ablation of renal masses measuring 3.0 cm and smaller: comparative local control and complications after radiofrequency ablation and cryoablation
13	10.1158/0008-5472.CAN-11-1782		21	Potent induction of tumor immunity by combining tumor cryoablation with anti-CTLA-4 therapy
14	10.1016/J.EURURO.2012.03.006		21	Focal cryotherapy for clinically unilateral, low-intermediate risk prostate cancer in 73 men with a median follow-up of 3.7 years
15	10.1016/J.JVIR.2009.12.403		20	Cryoablation: mechanism of action and devices
16	10.1111/J.1464-410X.2011.10578.X		20	Focal cryotherapy for localized prostate cancer: a report from the national Cryo On-Line Database (COLD) Registry
17	10.1016/J.EURURO.2015.03.027		20	Cryoablation for Small Renal Masses: Selection Criteria, Complications, and Functional and Oncologic Results
18	10.1016/J.EURURO.2016.08.044		20	New and Established Technology in Focal Ablation of the Prostate: A Systematic Review
19	10.1111/J.1464-410X.2005.05502.X		19	The molecular basis of cryosurgery
20	10.1148/RADIOL.10081634		19	Principles of and advances in percutaneous ablation
21	10.1016/J.CRYOBIOL.2013.11.001		18	Mechanisms of cryoablation: clinical consequences on malignant tumors
22	10.2214/AJR.15.14752		18	Renal Ablation Techniques: State of the Art
23	10.3174/AJNR.A4521		18	Spine Cryoablation: Pain Palliation and Local Tumor Control for Vertebral Metastases
24	10.1016/S0090-4295(02)01683-7		17	The cryobiology of cryosurgical injury
25	10.1016/J.JURO.2008.07.108		17	Best practice statement on cryosurgery for the treatment of localized prostate cancer
26	10.1089/END.2014.0881		17	Comparison of Outcomes Between Preoperatively Potent Men Treated with Focal Versus Whole Gland Cryotherapy in a Matched Population
27	10.1097/JTO.0000000000000632		17	Evaluating Cryoablation of Metastatic Lung Tumors in Patients--Safety and Efficacy: The ECLIPSE Trial--Interim Analysis at 1 Year
28	10.1016/J.JVIR.2015.02.010		17	Percutaneous cryoablation of stage T1b renal cell carcinoma: technique considerations, safety, and local tumor control
29	10.1016/J.JVIR.2014.12.006		17	Five-year survival after cryoablation of stage I nonsmall cell lung cancer in medically inoperable patients
30	10.1016/J.JVIR.2017.07.013		17	Percutaneous Cryoablation of Renal Tumors: Is It Time for a New Paradigm Shift?

DOI = digital object unique identifier, LCS_3 years = total of local citation score in 2018–2020, TI = title of literatures.

### 3.4. Analysis of publications sources

**Figure 6.**
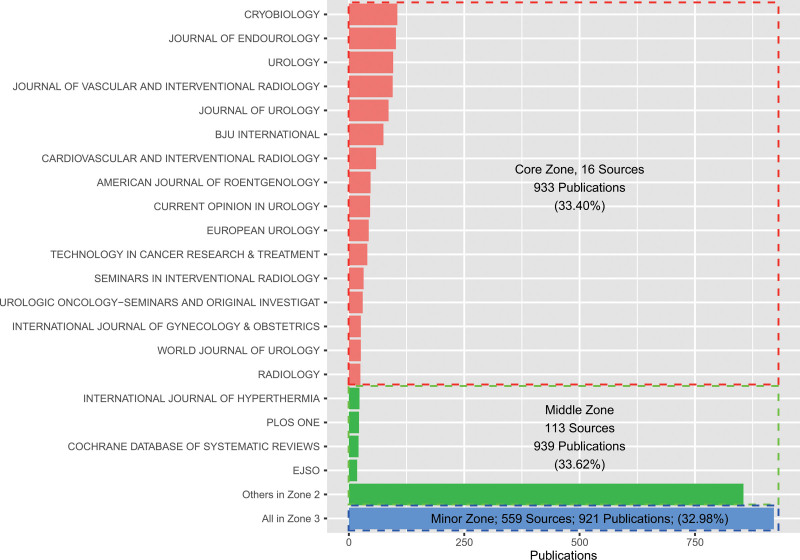
Analysis of publication sources according to Bradford law. The horizontal axis indicates the publication outputs of journals. The longitudinal axis indicates the names of journals.

**Figure 7.**
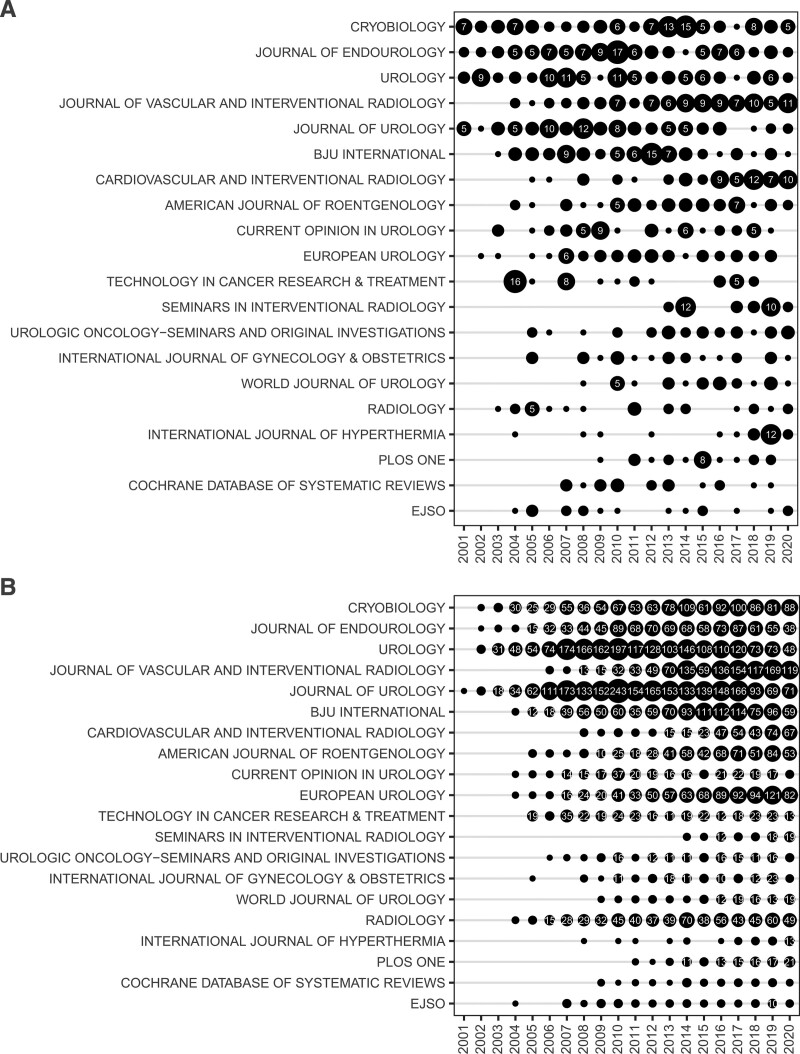
Actual annual publication outputs and LCS of the productive sources. Note: the size of black dots is proportional to actual annual publication outputs (a) or LCS (b) of the productive sources. Numbers inside black dots indicate actual annual publication outputs (a) or LCS (b) of the productive sources. LCS, local citation score. Productive sources refer to the publication sources in the core zone and the top 4 sources in the middle zone. LCS = local citation score.

In the present study, 688 publication sources were identified, of which 239 (34.74%) published 80% of the total publications within the cryoablation and neoplasm field (see Fig. S2, Supplemental Digital Content, http://links.lww.com/MD/I228, which illustrates the relationship between cumulative percent of publication outputs and publication sources). In addition, no more than 3 publications were published in each remaining source. According to Bradford law (Fig. 6), 16 publication sources were determined within the core zone. The top 5 sources were Cryobiology, Journal of Endourology, Urology, Journal of Vascular and Interventional Radiology (JVIR), and Journal of Urology. The middle and minor zones contained 113 and 559 publication sources, respectively. The publication sources in the core zone and the top 4 sources in the middle zone were classified as productive sources. Subsequently, the annual publication outputs and actual annual LCS of productive sources were calculated as indexes for evaluating productive sources. The annual publication outputs of *Cryobiology*, as a professional journal in the field of low-temperature biology and medicine, have steadily remained relatively high. The publication outputs of the JVIR and Cardiovascular and Interventional Radiology (CVIR) have increased since 2011 and 2015, respectively. A series of journals in the field of urology also published a large number of literature about cryoablation and oncology, but the annual publication outputs of each journal have declined in nearly 5 years (Fig. 7a). Moreover, almost all the productive sources had a steady academic influence, as observed from the actual annual LCS (Fig. 7b). In general, most of the productive sources have a higher LCS. However, *Radiology*, located at the bottom of the list of productive sources, had a higher LCS than some journals at the top of the list of productive sources. Moreover, changing trends of the journals in the field of urology regarding the actual annual LCS were similar to their changing trend of annual publication outputs.

## 4. Discussion

Cryotherapy is an old technique with relatively new innovations for treating neoplasms. Although cryogenic technology was used to treat breast and uterine tumors as early as the 1850s,^[23]^ it was not until the 1990s that cryotherapy made significant progress with the development of medical imaging technology and the application of the Joule–Thomson effect.^[24]^ More recently, cryoablation has been broadly used to treat tumors throughout the body. Given the wide range of cryoablation applications, it is challenging to identify important publications and themes in the field of cryoablation and oncology. The present study aimed to reveal the research status and focus in these fields from 2001 to 2020. To the best of our knowledge, this is the first study about the problem using a bibliometric approach.

Initially, the search strategies were performed in the topic domain, including title, abstract, author keywords, and *keywords plus*. Subsequently, considering that *keywords plus* introduces many publications unrelated to the target topic, despite the enhanced searching power with *keywords plus*,^[25]^ we performed a manual review of every publication retrieved by the search strategies (see Material and Methods section for details), with a filtering policy similar to the “front page” filter.^[26,27]^ Finally, 2793 publications involving cryoablation of neoplasm were identified. Overall, the increasing trend of cumulative publications from 2001 to 2020 was met with a growth linear model, although there were 2 small increases in annual publications from 2001 to 2012. It indicates that the number of cumulative publication growth in the research fields of cryoablation and oncology is relatively slow and that the number of new publications in this field annually reaches constant roughly. This result also suggests that existing theoretical bases in this field have been relatively mature.^[28]^

One of the main goals of this study was to explore the topics and frontiers in the fields of cryoablation and oncology. Five clusters were identified in the keyword co-occurrence networks that could more readily help researchers understand the landscape of cryoablation and oncology. Of these, clusters 1 to 4 represent studies of cryoablation in liver, lung, skin, prostate, and kidney cancers. There are some keywords with similar meanings in these subclusters, such as “follow-up,” “risk,” “efficacy,” “survival,” “long-term outcome,” “outcome,” and “complication.” Moreover, it can be assumed that studies in these fields are concerned with the safety and efficacy of tumor cryotherapy, with only differences in tumor locations. In addition, in the subfields of liver, lung, and kidney cancers, researchers have focused on the intercomparison between cryoablation and surgery, such as partial or laparoscopic partial resection. In the subfield of skin cancer, researchers have focused on the intercomparison between cryoablation and photodynamic therapy. In the field of prostate cancer, in addition to surgery, researchers have also focused on the intercomparison between cryoablation and radiotherapy, short-course radiotherapy, complications, and HIFU. Cluster 5 represents studies of cryoablation versus other energy ablation therapies in various types of solid tumors, with investigators focusing on the efficacy and safety of cryoablation versus RFA, MWA, or IRF. More recently, Kim and Kwak *et al* discussed the current status of cryoablation in various solid tumor types, which were similarly categorized by liver, kidney, prostate, and skin cancers.^[29]^

Each bubble in the keyword strategy coordinate diagram represents a research topic, and the topic’s label is the representative keyword in that topic. The horizontal coordinate of the coordinate chart is the centrality, which reflects the connection between research topics. The vertical coordinate is the density, which represents the connection between unit knowledge points within the research topic.^[18]^ The thematic map clearly shows the changes in research themes in the field of tumor cryoablation over the past 20 years and predicts future research hotspots to some extent. In the thematic map for the period from 2001 to 2012, the research topics with representative keywords of “injury,” “tissue,” “model,” and “animal model” are located in the upper right quadrant of the keyword thematic map, which represents major topics in the field of tumor cryoablation. During this period, researchers have focused on the mechanism of direct tissue damage by cryogenic temperature and the effects of ice sphere shape, range, freezing time, several freeze-thaw cycles, and peri-lesion tissue on the freezing effect.^[30–38]^ Compared with *ex vivo* models, animal models can better meet experimental requirements and help investigators optimize these factors before conducting human trials. In the thematic map for the period from 2013 to 2020, the representative keywords of this research theme, in addition to “animal model,” shift to “immunotherapy” and “chemotherapy.” An increasing number of research results also show that cryoablation can have a systemic antitumor effect in synergy with immunotherapy and chemotherapy, in addition to local cytotoxic effects.^[39–44]^ This suggests that the focus of investigators is gradually shifting toward the synergistic effects of cryoablation in combination therapy. Over time, this evolution of representative keywords is also observed in the research themes represented by prostate cancer and radiotherapy. After 2013, HIFU became a new representative keyword under this research theme, suggesting that the role played by HIFU in prostate tumors is gradually gaining attention from urology researchers.^[45]^

Two completely new research themes also appeared in the thematic map for the period from 2013 to 2020. First, new research themes with the representative keywords “high-grade dysplasia,” “Barrett’s esophagus,” “spray cryotherapy,” “efficiency,” and “safety” were located between the upper and lower left quadrants of the coordinate chart. Barrett’s esophagus is considered a precancerous lesion to esophageal cancer and is an intestinal metaplasia that gradually evolves from low-grade to high-grade heterogeneous hyperplasia and eventually to intramucosal carcinoma.^[46,47]^ The current treatment of choice for Barrett’s esophagus is RFA, and other treatment options include endoscopic mucosal resection, endoscopic mucosal dissection, photodynamic therapy, and surgery.^[48]^ Cryoablation is a relatively new treatment method, and its effectiveness and safety have gradually been recognized by researchers.^[49,50]^ Compared with RFA, cryoablation has fewer side effects and can still achieve good results in patients who have failed RFA.^[51]^ However, high-quality clinical studies remain significantly limited, and cryoablation has not been recommended as a first-line treatment option for Barrett esophagus (highly heterogeneous hyperplasia). Still, it is assumed to likely be a popular research topic in the future. Second, a new research theme with “retinoblastoma” and “arterial infusion” as representative keywords has emerged in the niche theme quadrant. Retinoblastoma is the most common ocular malignancy in children. In the past, eye removal was often used as the standard of care for advanced retinoblastoma,^[52]^ but in the last 30 years, eye preservation has gradually become a new requirement. Transarterial infusion chemotherapy has been increasingly used in the last decade because of its remarkable efficacy in eye preservation.^[53–55]^ However, cryotherapy is now rarely used alone in treating retinoblastoma but is more often used in combination with transarterial infusion chemotherapy. Therefore, this research theme with “retinoblastoma” and “arterial infusion” as representative keywords may not represent a popular topic in the field of tumor cryoablation but is rather an incidental manifestation of the increasing research interest in transarterial infusion chemotherapy in retinoblastoma. This also reminds researchers that judging research hotspots based solely on keyword analysis may lead to misinterpretation of results.

Important literature within a research field can help researchers gain a more detailed understanding of the research hotspots within the field. The importance of publications is determined by the number of its citations.^[56]^ Although the number of publication citations is not the only indicator of the publication’s quality assessment, to some extent, it often reflects the impact of the publication, regardless of whether it is positive or negative.^[7]^ The present study initially listed the top 30 most important publications based on annual average LCS, with cryoablation for renal and prostate cancers accounting for most studies. This is consistent with the results of the keyword co-occurrence network of the present study, indicating that these topics have been receiving remarkable attention from the academic community for the last 20 years. In addition to the literature on these 2 topics, the list mostly comprises reviews on the molecular biological basis and clinical applications of cryoablation. Such types of literature help researchers understand the basis and status of research in the field of cryoablation of tumors; however, they do not reflect the currently popular research topics in the field well. Researchers should pay more attention to the literature with more recent citations rather than just to the literature with higher total LCS or total citation frequency because of the age of the literature.^[57]^ The graph of actual citations per year in this study also provides a visualization of the life cycle of the literature and the aging phenomenon. Therefore, the present study further counted the top 30 articles regarding total LCS in the last 3 years (2018–2020). The results showed that cryo-immunology: a review of the literature and proposed mechanisms for stimulatory versus suppressive immune responses ranked in the top 5, both in terms of average annual and total LCS over the last 3 years, and the article Potent induction of tumor immunity by combining tumor cryoablation with anti-CTLA-4 therapy was also ranked in the top 30 of the total LCS for the last 3 years. These results indicate that the immunomodulatory mechanism of cryoablation has gradually become a popular research topic in the field. This conclusion is also validated in the thematic map presented in the present study. Although the immunomodulatory phenomenon of cryoablation has been reported as early as the 1960s,^[58]^ the immunomodulatory mechanism of cryoablation has received increasing attention from researchers, given that immunotherapy plays a crucial role in tumor treatment. Recently, a growing number of preclinical and phase I/II clinical studies have shown that cryoablation in conjunction with immune checkpoint inhibitors has promising applications in treating malignancies.^[41,59–61]^ A study by Campbell *et al* demonstrated that tremelimumab in combination with cryoablation is feasible for treating metastatic renal clear cell carcinoma and that cryoablation can modulate patients’ immune microenvironment.^[60]^ Results from a prospective, proof-of-concept cohort study by Fan and Shen *et al* also showed a significant increase in the number of natural killer cells and a decrease in the number of regulatory T cells observed during cryoablation with a continuous arterial infusion of pembrolizumab for liver metastases of melanoma.^[61]^ More in-depth basic and clinical studies under this research theme are expected in the future.

The results of the bibliometric analysis of important literature publications can identify research hotspots that are difficult to discover by keyword analysis alone. Cryoablation of bone tumors as a separate category was hard to clearly reflect in the keyword co-occurrence network and the keyword thematic map. However, the statistical results of annual mean LCS, total LCS in the last 3 years, and annual actual LCS showed that 2 clinical studies on cryoablation for bone metastases entered the list of important literature. Additionally, no signs of aging in the literature were observed in both of them, suggesting that using cryoablation to control intractable pain caused by bone metastases has also been a focus in recent years. Both a 2013 single-arm multicenter clinical trial on cryoablation for painful bone metastases and a 2016 retrospective clinical trial on cryoablation for vertebral metastases suggest that cryoablation is safe and effective for refractory pain due to bone metastases.^[62,63]^ The new 2021 MOTION multicenter clinical study further increases the evidence for cryoablation as a first-line option for treating painful bone metastases. It once again demonstrates the popularity of this research topic.^[64]^

Identifying important journals in the field can help improve the efficiency of researchers in searching and tracking research results. Although the impact factor (IF) is often used to evaluate the impact of journals, it is more appropriate to compare the impact of journals in the same research area, given that IF is calculated as the average number of citations per paper published in the journal in the previous 2 years.^[65]^ As an interventional treatment, cryoablation is inherently interdisciplinary; therefore, journals in the field of tumor cryoablation may not be suitable for evaluation and comparison using IF. In the present study, 20 important journals were identified based on Bradford law and were further evaluated by their actual annual article counts and annual LCS. *Cryobiology* is a specialty journal in the field of cryobiology and is at a high level in terms of both volume and annual LCS. Additionally, some important literature on the mechanism of cryoinjury is published in this journal, making it one of the journals of interest for understanding the field of tumor cryoablation. The *JVIR* is also an important journal in the field of cryoablation of tumors, and its annual volume has been high since 2012. In the list of the top 30 important papers in the total LCS in the last 3 years, several papers have been published in the *JVIR*, and possibly, important results in the field of tumor cryoablation will appear in this journal in the future. In addition, *CVIR* is also an important journal in the field; however, there are fewer publications from *CVIR* in the current list of significant literature, but this may be because the number of publications in the field of tumor cryoablation in this journal has been gradually increasing since 2015. Although *Radiology* is an established journal in the field of diagnostic radiology, its annual LCS is high, indicating that researchers of interventional oncology recognize its publications. For example, the 2014 article Image-guided tumor ablation: standardization of terminology and reporting criteria—a 10-year update defines the terminology and reporting criteria for image-guided ablation therapy that are still widely used today.^[66]^ In addition, since cryoablation is widely used in kidney and prostate cancers, there are many urology journals among the important journals categorized, and care should be taken not to overlook this section when tracking the literature related to the cryoablation of urological tumors.

This study has some limitations. First, the bibliometric analysis targets published literature, which may overestimate earlier publications and underestimate more recent ones. This study calculated the annual average LCS of the literature, actual LCS for each year, and total LCS for the last 3 years to avoid the effect of the year of publication on the results. However, some newly published literature is ignored because it has not yet accumulated sufficient citations^.[67]^ To control this, one study has suggested that at least a decade of citation accumulation is required to assess the impact of papers.^[68]^ Second, previous studies have argued that the centrality of a research topic in a thematic map represents the importance of that topic within the overall research field. However, the centrality of each research topic in this paper may not accurately reflect its position in the overall research field but rather reflects the heterogeneity among various types of tumors. Future refinement of the cancer field and repeated bibliometric analysis and acquisition can result in more detailed information. Finally, the purpose of this study was not to review the quality of the literature or the level of evidence of the proposed findings, nor is it intended to compare the level and quality of research by researchers, journals, and countries in the field, as these require a more comprehensive multiparametric evaluation.

## 5. Conclusion

In this study, we used a bibliometric approach to assess the global research dynamics in the field of cryoablation of tumors over the last 20 years (2001–2020). The number of new papers in the field has been relatively constant in recent years. Research topics in this field include the clinical application of cryoablation in liver, lung, kidney, prostate, and skin tumors and comparing cryoablation with other energy ablations in several solid tumors. Cryoablation, combined with immunotherapy, has gradually become a popular research topic. The application of cryoablation in bone metastases and endoscopic cryospray treatment has also attracted the attention of some investigators. In addition, the study identifies several important articles and journals in the field, which helps researchers rapidly comprehend the focus in the field and develop an appropriate literature tracking strategy.

## Acknowledgments

We are grateful to Professor Yunlong Yu for his course in bibliometrics. We are also grateful to the professional editors at Editage, who provided us with excellent assistance in the production of this manuscript.

## Author contributions

**Conceptualization:** Hang Liu, Jijin Yang.

**Data curation:** Hang Liu, Changen Song, Jijin Yang.

**Formal analysis:** Hang Liu, Changen Song, Rong Luo.

**Funding acquisition:** Jijin Yang.

**Methodology:** Changen Song, Bingzhe Zhang, Rong Luo, Jijin Yang.

**Supervision:** Jijin Yang.

**Visualization:** Hang Liu, Bingzhe Zhang.

**Writing—original draft:** Hang Liu, Changen Song.

**Writing—review and editing:** Hang Liu, Changen Song, Bingzhe Zhang, Rong Luo, Jijin Yang.

## Supplementary Material
