# Supplementary material for: Research trends and areas of focus on cryoablation and oncology: A bibliometric analysis from 2001 to 2020
Source: Medicine (Baltimore). 2022 Dec 30;101(52):e32513. doi: 10.1097/MD.0000000000032513 (PMC9803458; doi:10.1097/MD.0000000000032513)

Fig. S1 Distribution pattern of all keywords. The horizontal axis indicates the cumulative percent of keywords. The longitudinal axis indicates the cumulative percent of keyword frequency.

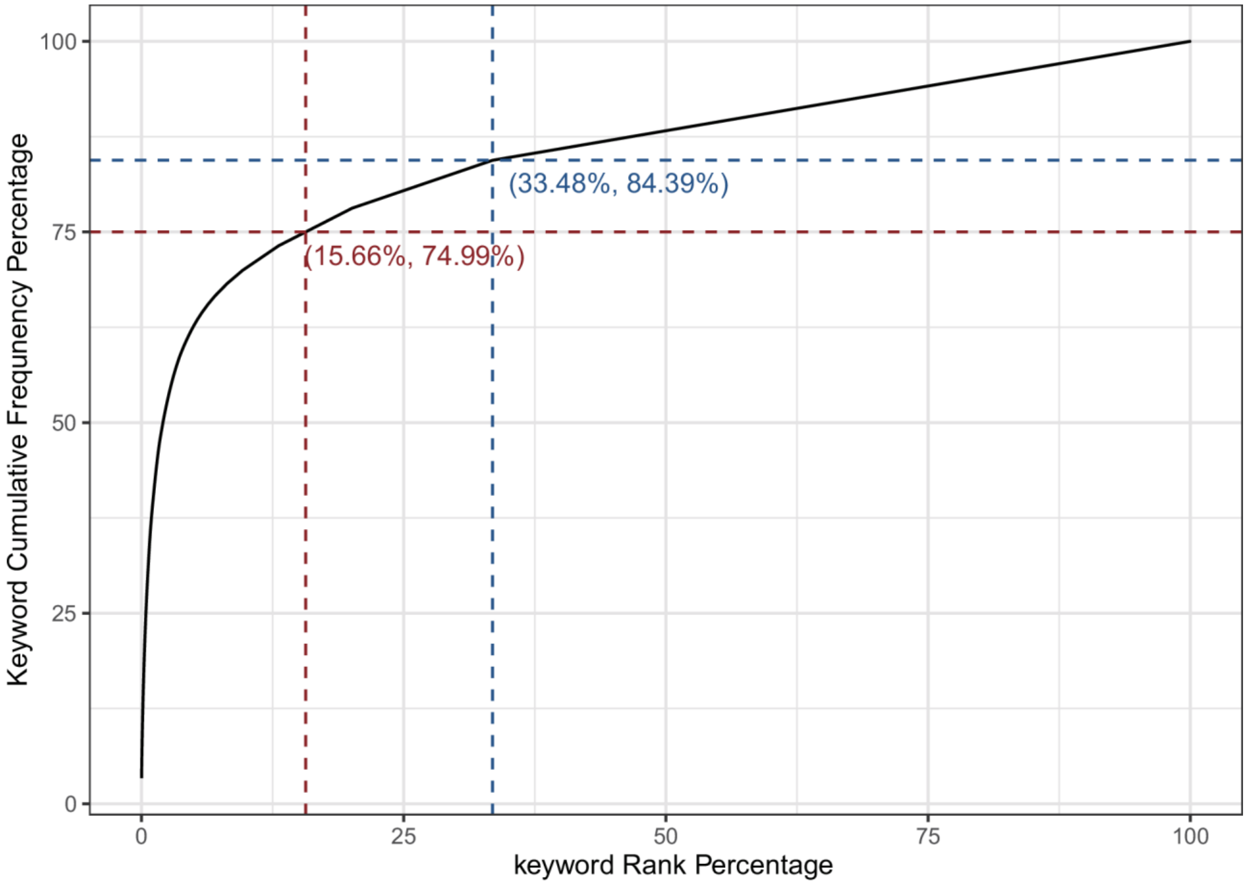

Supplement: Supplementary file 1 [file medi-101-e32513-s001.pdf]
