# Supplementary material for: Research trends and areas of focus on cryoablation and oncology: A bibliometric analysis from 2001 to 2020
Source: Medicine (Baltimore). 2022 Dec 30;101(52):e32513. doi: 10.1097/MD.0000000000032513 (PMC9803458; doi:10.1097/MD.0000000000032513)

Fig. S2 Relationship between cumulative percent of publication outputs and publication sources.

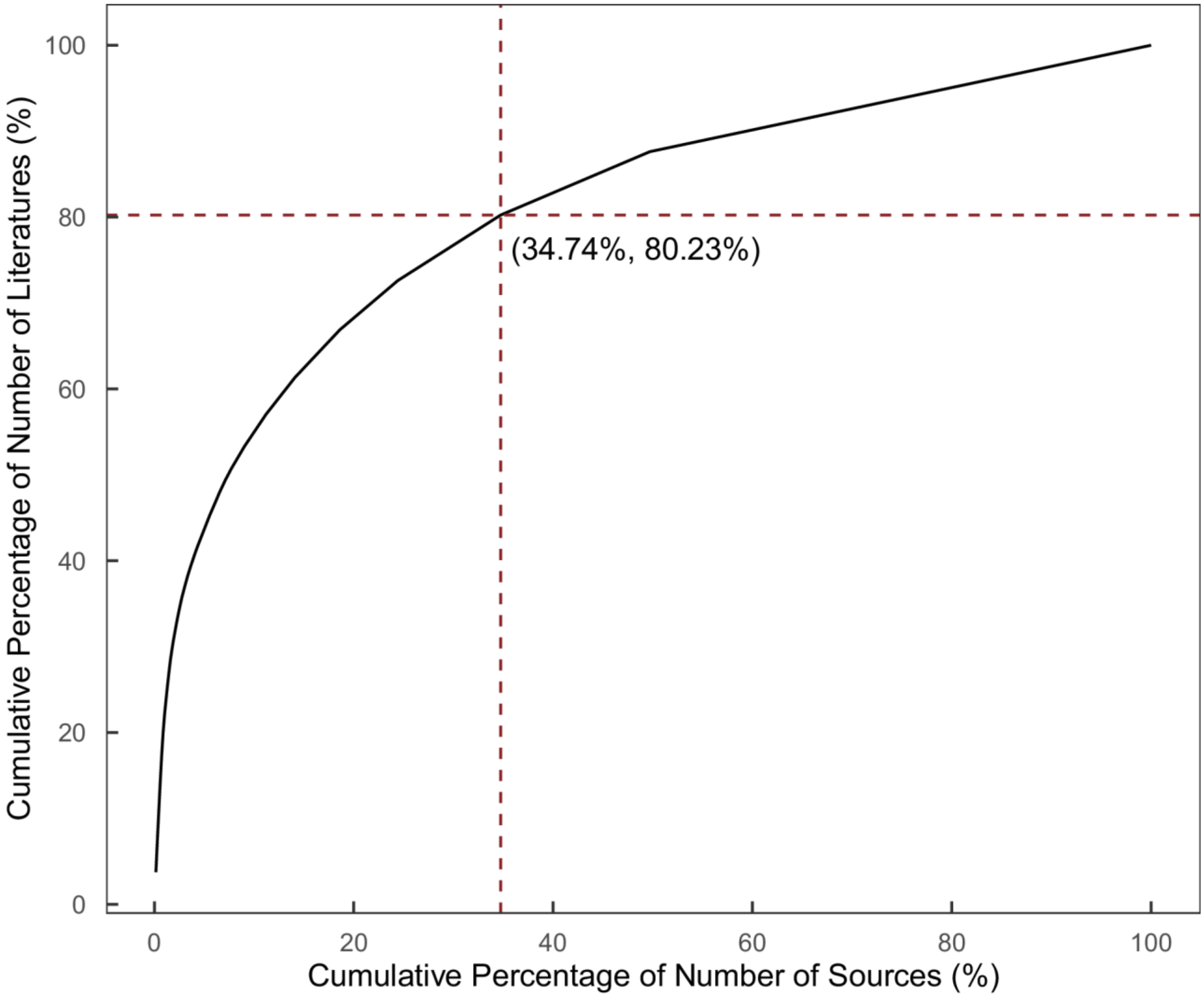

Supplement: Supplementary file 2 [file medi-101-e32513-s002.pdf]
